# Characterization of the Sortase Repertoire in *Bacillus anthracis*

**DOI:** 10.1371/journal.pone.0027411

**Published:** 2011-11-04

**Authors:** Willy Aucher, Sophie Davison, Agnès Fouet

**Affiliations:** 1 Institut Pasteur, Unités Toxines et Pathogénie Bactérienne, Pathogénèse des Toxi-infections Bactériennes, Département de Microbiologie, Paris, France; 2 CNRS URA 2172, Paris, France; 3 Insitut Cochin, Team FRM "Barriers and Pathogens", INSERM U-1016, Paris, France; 4 CNRS UMR-8104, Paris, France; 5 Université Paris Descartes, Sorbonne Paris Cité, Paris, France; Science and Technology Facilities Council, United Kingdom

## Abstract

LPXTG proteins, present in most if not all Gram-positive bacteria, are known to be anchored by sortases to the bacterial peptidoglycan. More than one sortase gene is often encoded in a bacterial species, and each sortase is supposed to specifically anchor given LPXTG proteins, depending of the sequence of the C-terminal cell wall sorting signal (cwss), bearing an LPXTG motif or another recognition sequence. *B. anthracis* possesses three sortase genes. *B. anthracis* sortase deleted mutant strains are not affected in their virulence. To determine the sortase repertoires, we developed a genetic screen using the property of the gamma phage to lyse bacteria only when its receptor, GamR, an LPXTG protein, is exposed at the surface. We identified 10 proteins that contain a cell wall sorting signal and are covalently anchored to the peptidoglycan. Some chimeric proteins yielded phage lysis in all sortase mutant strains, suggesting that cwss proteins remained surface accessible in absence of their anchoring sortase, probably as a consequence of membrane localization of yet uncleaved precursor proteins. For definite assignment of the sortase repertoires, we consequently relied on a complementary test, using a biochemical approach, namely immunoblot experiments. The sortase anchoring nine of these proteins has thus been determined. The absence of virulence defect of the sortase mutants could be a consequence of the membrane localization of the cwss proteins.

## Introduction

Proteins can be covalently attached to the peptidoglycan of Gram-positive bacteria and this binding reaction is catalyzed by membrane-associated transpeptidases called sortases (for review, [1], [2], [3]). The anchored proteins contain a characteristic carboxyterminal sorting signal consisting of a so-called LPXTG motif followed by a hydrophobic domain and a positively charged tail, leading to a transiently membrane associated protein [4]. The enzymatic activity of sortases can be defined as a cleavage between the threonine and the glycine amino acid of the LPXTG motif to generate an acyl-enzyme intermediate which then reacts with an N-terminal amino-acid on the lipid II, regenerating an amide bond. Alternatively, the acceptor may be a protein [1], [5], [6], [7]. Sortase genes are ubiquitous among Gram-positive bacteria. The number of different sortase genes present in a given genome depends on the species [8]. The vast majority of the studied species have between two and seven sortase genes [9]. Sortases from Gram-positive species can be classified into five or four homology groups [2], [3], [9]. Sortase A is the major sortase, present in the genomes of all Gram-positive bacteria. The other sortases seem to play more defined roles: B sortases anchor proteins involved in iron acquisition, C sortases are involved in pilus polymerization as do B sortases sometimes, and may be required for adaptation to specific niches [10], [11], [12], [13] and D sortases are harbored by species displaying some specific developmental cycle. Therefore, the sortases B, C and D may be expressed at specific times in the bacterial life cycle [2].

This profusion of sortases in bacterial genomes raises questions about sortase repertoires. Previous studies have indicated that sortases recognize their protein substrates specifically and cannot anchor other sortase substrates; it is thus possible to establish theoretical sortase repertoires [9]. However, some motifs appear ambiguous. For example, proteins containing the LPETG or LPKTG motifs may be attributed to the sortase A or sortase C repertoire. In addition, some motifs do not resemble any of the identified consensus sequences. For example, a QVPTG motif-harboring protein in *S. pyogenes* is a sortase C2 substrate, a class B sortase involved in pilus assembly [14]. Furthermore, when this QVPTG motif is replaced by the LPSTG of a *S. pyogenes* sortase A-anchored protein, sortase A is able to cleave the LPSTG motif, but neither sortase A nor sortase C2 can polymerize the chimera [14]. In that case, the cell wall sorting signal (cwss) is sufficient to determine the specificity of cleavage, but transpeptidation of the intermediate requires additional signals. Thus, the motif is not sufficient to determine sortase specificity.

We report here an analysis of *B. anthracis* cwss proteins and sortase repertoires. *B. anthracis* is the etiological agent of anthrax (for review, [15]). To note, *B. anthracis* also possesses an anchoring mechanism by which proteins harboring a SLH domain are non-covalently anchored to the peptidoglycan by means of binding to a pyruvylated polysaccharide [16]. *B. anthracis* cwss proteins will hereafter refer to proteins that are covalently anchored to the peptidoglycan by the mechanism involving sortases. Based on its genome sequence, this bacterium appears to have three sortases (Srt) — a class A sortase, SrtA, the structure of which has recently been shown to differ from that of other SrtAs [17], a class B sortase, SrtB, and a class D sortase known, and referred to hereupon, as SrtC [18]—and 9 to 11 putative cwss proteins, depending on the analyzed strain [9], [19], [20]. Interestingly, the triple *srtA srtB srtC* mutant strains, deleted for all three sortase genes, are not affected in their virulence, independently of the genetic background, toxinogenic or encapsulated [21], [22].

The *in vitro* cleavage of synthetic peptides by sortases and the *in vivo* cleavage and anchoring of some entire cwss proteins in *B. anthracis* have been reported. A model peptide harboring the KTDNPKTGDEA sequence from the IsdC protein was found to be cleaved only by SrtB *in vitro* [23]. Similarly, a peptide containing the LPATG motif (as found in BasC) was found to be cleaved by SrtA and a peptide containing the LPNTA motif (as present in BasH and BasI) was found to be cleaved by SrtC only or, to a lesser extent, also by SrtA, depending on the experimental conditions [18], [20]. Thus, *B. anthracis* sortases may display different degrees of specificity for the *in vitro* cleavage of some peptides. These findings may be a good indication of sortase cleavage specificity, but cannot predict the ability of the sortases to anchor the corresponding protein to the peptidoglycan.

The anchoring of complete proteins in *B. anthracis* has been studied. SrtA and SrtB anchor BasC and IsdC, respectively [20], [23]. SrtC and BasI are encoded by genes in the same operon, which is maximally expressed at the onset of the stationary phase [24] and under the control of SctR, a transcriptional activator [18]. SrtC mediates the anchoring of BasI to the peptidoglycan of the sporulating bacillus. BasH, which is expressed in the forespore, may be anchored to the primordial peptidoglycan of the spores by sortase C. GamR, also termed BasD, a *B. anthracis* γ phage receptor, is anchored by SrtA. A GamR mutant strain is resistant to γ phage lysis and an *srtA* mutant strain displays attenuated susceptibility to this phage, when added at high titer, and is resistant in presence of lower phage titers [25]. Furthermore, double *srtA srtB* or *srtA srtC* and the triple mutant strains displayed the same resistance/susceptibility phenotype as the *srtA* mutant, whereas the double *srtB srtC* strain was fully sensitive to γ phage lysis. These data indicate that GamR is anchored by SrtA [25]. There are conflicting results, concerning IsdX2 also termed IsdK, that may or not be in part covalently anchored to the peptidoglycan [26], [27].

*B. anthracis* SrtA structure indicates that an N-terminal extension may affect how lipid II is recognized [17]. Furthermore, SrtA is unable to anchor an LPETG peptide to m-DAP, to which the cwss proteins are bound. In contrast to what is observed in other sortases, *B. anthracis* sortases may require additional protein components or larger portions of the lipid II to catalyze the transpeptidation reaction. Consequently, *in vivo* experiments to define the repertoires of the sortases may turn out to be necessary.

A genetic screen for the analysis of *in vivo* anchoring by sortases was devised, making use of the GamR function. Chimeric proteins harboring part of GamR and the cwss, preceded by a minimum of ten amino-acid residues, of each of the putative cwss proteins were produced in strains mutant for GamR and for sortases; the susceptibility of these strains to γ phage lysis was determined. Immuno-blotting experiments were used for chimeric proteins where the sensitivity of the screen led to ambiguous results. Predicted sortase repertoires were confirmed and the consequences of the presence at the cell surface of non-anchored cwss proteins are discussed.

## Materials and Methods

### Bacterial strains, plasmids and growth conditions

*Escherichia coli* TG1 [28] *E. coli* HB101 harboring pRK24 were used for cloning and mating experiments [29]. The *B. anthracis* strains and plasmids used are listed in Table S1. GamR expression under its native regulatory signals was obtained after growing cells in BHI (Difco). *pagA*-mediated *gamR* expression was induced by culturing bacteria on BHI plates, for susceptibility tests, and in R medium, for peptidoglycan purification, both supplemented with 0.6% bicarbonate, under an atmosphere containing 5% CO_2_ (BHI-bic and R-bic) [30], [31]. Antibiotics used were spectinomycin (100 µg/ml), kanamycin (40 µg/ml) and erythromycin (for *E. coli* 150 µg/ml; for *B. anthracis* 5 µg/ml).

### Bacteriophage and susceptibility test

γ phage (laboratory stock) was amplified as described elsewhere [25]. Exponentially growing cultures of *B. anthracis* cells were spread on plates and 20 µL of a 10^-2^ dilution of the γ phage solution were spotted onto the surface. Plaques were observed after 16 h of incubation at 37°C.

### Construction of recombinant strains

Recombinant strains were constructed as previously described [29], [32]. Transduction between *B. anthracis* strains was mediated by phage CP51 [33].

### DNA manipulation

Plasmid extraction, endonuclease digestion, ligation and agarose gel electrophoresis were carried out as described by Maniatis *et al.* [34]. Polymerase chain reaction (PCR) amplifications were carried out with long-range high-fidelity Taq polymerase (Roche), or with Vent polymerase (Biolabs), according to the manufacturer's instructions. Plasmid sequences, carried out to check for the absence of mutations on cloned fragments, were determined from PCR products after an amplification step or directly from the plasmid DNA. All sequencing was carried out by Genome Express Sequencing.

### Mutant constructs

The *gamR*-deleted strain was constructed as follows. pGARK20 was obtained by replacing the 1.1-kb Bcl1-Cla1 fragment in pGAR10, with a kanamycin resistant cassette extracted from pAT21 [25], [35]. The Δ*gamR* construction was excised from pGARK20, by digesting with SphI-EcoRI and inserted into pATΔS28, yielding pGARK30. The wild-type *gamR* allele was replaced with that in pGARK30 after conjugation and a double cross over event into the 7702 strain, giving rise to the 7AG strain. The *gamR srtA*, *gamR srtB*, and *gamR srtC* double mutants were obtained by transduction of the *gamR* deletion into 7SBON30, 7SBTR30 and 7SBTO30, respectively [25].

### Fusion constructs

**Figure 1:**
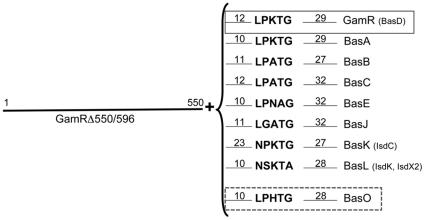
Schematic representation of native GamR and 8 chimeric proteins. The sequence encoding GamR from amino-acid one to 550 was fused to each of the 7 “cwss tails”; these are composed of 10 to 23 residues preceding the “cwss” motif, the motif and the native C-terminal end of the studied proteins. Similar constructs with the wild-type GamR and the chimeric protein harboring the pseudo-cwss tail of the non-cwss protein BasO are also represented and highlighted by a rectangle. Numbers indicate number of residues.

DNA fragments carrying the cwss-encoding end of the *basA, basB, basC, basE*, *gamR, basF, basG, basJ, basL* and *basO* (Fig. 1) genes were obtained by PCR amplification from *B. anthracis* 7702 chromosomal DNA, using the primers basA-5′ and basA-3′, basB-5′ and basB-3′, basC-5′ and bas C-3′, basE-5′ and basE-3′, GamR5′ and GamR 3′, basF-5′ and bas F-3′, basG-5′ and bas G-3′, basJ-5′ and bas J-3′, IsdC-5′ and IsdC-3′, basL-5′ and bas L-3′, basO-5′ and bas O-3′, respectively (Table S1).

The GamR-5′ primer contains an NdeI site overlapping the ATG initiation codon of the *gamR* gene. All other 5′ primers start with an AgeI site, positioned so as to allow translational fusion with the *gamR* gene, which contains an AgeI site.

The DNA fragments amplified were inserted into pCR2.1, giving pBasA10, pBasB10, pBasC10, pGamR10, pBasE10, pBasF10, pBasG10, pBasJ10, pIsdC, pBasL10, pBasO10, respectively.

The digestion of pGamR10 by AgeI/XbaI removed the LPXTG tail sequence from *gamR* (nt 1646 to nt 1787, codon 1 corresponding to A of the initiation codon). The resulting construct was used for further fusion constructions, in which the LPXTG-charged *gamR* tail was replaced by the tail of the other 10 proteins.

We obtained pBasA20, pBasC20, pBasL20 by inserting the AgeI/XbaI fragment from pBasA10, pBasC10, pBasH10, pBasL10 into pGamR10 digested with AgeI/XbaI.

pBasB20, pBasE20, pBasJ20, pIsdC20 and pBasO20 by inserting the Age1/Sac1 fragment from pBasB10, pBasE10, pBasI10, pBasJ10, pIsdc10 and pBasO10 respectively into pGamR10 digested with AgeI/SacI.

pBasA30, pBasC30 and pBasL30 by inserting the NdeI/XbaI fragment from pBasA20, pBasC20, pBasL20, respectively, into pPPA40 digested with NdeI/XbaI [36].

We obtained pBasB30, pBasE30, pBasJ30, pIsdC30 and pBasO30 by inserting the NdeI/SacI fragment from pBasB20, pBasE20, pBasJ20, pIsdc20, and pBasO20, respectively, into pPPA40 digested with NdeI/SacI.

We obtained pGamR30 by inserting the NdeI/SacI fragment from pGamR10 into pPPA40 digested with NdeI/SacI.

### Complementation constructs

The *B. anthracis* strains harboring a plasmid encoding the chimeric protein and deletions of the *gamR* gene and the *srtA* gene were already resistant to the three antibiotics currently available in our laboratory. The wild-type *srtA* gene was therefore inserted directly into the plasmid carrying the *gamR* fusion gene. We ensured SrtA synthesis by creating a new operon, by inserting the *srtA* gene downstream from the *gamR* fusion gene stop codon. The DNA fragment carrying the sortase A gene was obtained by PCR amplification from *B. anthracis* 7702 chromosomal DNA, using the primers CA1-5′/CA2-3′. The 5′ ends contain a SacI restriction site followed by a sequence harboring a RBS — GGAGGAAAAATATAA — immediately upstream from the ATG initiation codon of the sortase gene. The fragment was inserted into pCR2.1, giving pCA10. The sortase gene was excised by digestion with EcoRI-SacI and blunted. The *srtA* gene was inserted into pBasB30, pBasE30 and pBasJ30 digested with EcoRI and blunted, giving pBasB40, pBasE40, pBasJ40, respectively.

The fusion and complementation constructs were transferred by mating experiment into the 7G, 7AG, 7BG and 7CG strains.

### GamR production construct

To produce GamR, *gamR* gene was inserted into pQE30 as follows. *gamR* was amplified using Bas5′ and Bas3′ and inserted into pGEM-T-easy, yielding p3367. p3367 was digested by BamHI-HindIII and ligated into pQE30 similarly digested, giving rise to pQE3367.

### Cell fraction preparation

Cell fractionation was carried out as previously described [37]. Briefly, *B. anthracis* liquid cultures were centrifuged; supernatant fraction was precipitated with 10% TCA, pellets were sonicated and the soluble fraction (corresponding to the cytoplasm) and insoluble fraction (corresponding to membrane and peptidoglycan fractions) were separated.

### Peptidoglycan preparation

Peptidoglycan was prepared essentially as described by [37]. Briefly, cells were grown in 30 mL of R-bic to an OD_600_ of 1. Ten mL of the supernatant were TCA precipitated. After each subsequent treatment, an appropriate centrifugation was carried out. The pellet was suspended in 5 mL of 50 mM Tris-HCl pH 7.4, 150 mM NaCl, 1% SDS and boiled for 10 min and 1 mL was put aside (step 1) and the rest centrifuged. The resulting pellet was resuspended in 4 mL and treated similarly; again 1 mL was kept (step 2). The final pellet was resuspended in 3 mL of the same buffer and sonicated twice 30 s (step 3).

The one mL fractions, kept at each step, were centrifuged. The pellets rinsed with 1 mL of the Tris NaCl, buffer, resuspended in 100 µL containing 10 units of mutanolysin and digested for 2.5 h at 37°C. Volumes corresponding to the same percentage of the initial pellet were loaded on SDS-gels.

### Antibody production

*Escherichia coli* M15/pREP4/pQE3367 was cultivated and His-tagged GamR was purified as described by Qiagen on Ni-NTA resin as described by Qiagen. GamR serum was obtained by subcutaneously injecting 10 µg of His-tagged GamR in Freund incomplete adjuvant into OF1 mice (Charles River) four times at 2-week intervals.

### Immunodetection

For the Western blot analyses, samples were separated on SDS-12% polyacrylamide gels and the proteins transferred onto nitrocellulose membranes. GamR or chimeric proteins were detected using anti-GamR antibodies diluted at 1/650.

## Results

### *In silico* analysis

Our *in silico* study suggested the existence of 10 cwss proteins in the Ames genome [38] (Table 1). We also found 4 other proteins with LPXTG motifs but unconvincing hydrophobic tails, including that termed BasO. Based on this *in silico* study and prediction methods [9], we suggested the most likely sortase involved in anchoring these 10 proteins; we assigned the same sortases as Gaspar [20] for 8 proteins and SrtA for BasJ and SrtB for BasL/IsdK/IsdX2 protein (NSKTA motif) (Table 1).

**Table 1:** *B. anthracis* putative cwss proteins and their anchoring sortase.

Protein name(s)	Ames number^a^	cwss motif	predicted anchoring sortase	*In vitro* peptide cleavage^b^	*In vivo* anchorage^c^
BasA	BA4346	LPKTG	SrtA		
Bas B	BA0871	LPATG	SrtA		
Bas C	BA5258	LPATG	SrtA	[20]	NI-NTA chromatography after expression of BasC histidine fusion [20]
GamR/BasD	BA3367	LPKTG	SrtA		Gamma phage sensitivity [25]
BasE	BA3254	LPNAG	SrtA		
BasH	BA0397	LPNTA	SrtC	[18]	Fluorescent microscopy after expression of fusion proteins [18], [47]
BasI	BA5070	LPNTA	SrtC		
BasJ	BA0552	LGATG	SrtA		
BasK/IsdC	BA4789	NPKTG	SrtB	[23]	Cell wall localization after fractionation of an IsdC histidine fusion [23]
BasL/IsdK, IsdX2	BA4787	NSKTA	SrtB		Cell associated localization after crude fractionation [26]
BasO	BA1088	LPHTG	NA		

a TIGR.org.

b :2-aminobenzoyl-LPXTG motif-diaminoprpionic acid was incubated with purified sortase and cleavage is measured by fluorescence.

c :*in vivo* anchoring of native, GamR, or GFP or histidine fusion proteins, BasC, BasH, BasI, IsdC, by different techniques.

NA; does not apply, no hydrophobic tail.

### Cwss protein identification

In a previous study, we identified the *B. anthracis* γ phage receptor, GamR, an LPXTG protein anchored by SrtA [25]. We investigated whether the proteins selected during our *in silico* study were true cwss proteins, by constructing GamR fusions, in which the cwss tail of GamR was deleted and replaced with “cwss tails” of 8 other proteins, 7 putative cwss proteins and the non-cwss protein BasO, starting at least 10 amino-acid residues before the cwss motif and extending to the native C-terminal end of the studied proteins. (Materials and Methods, Fig.1). The fusion proteins were cloned downstream from the *pagA* promoter and translation initiation site. Their expression is induced in presence of bicarbonate and CO_2_ [31].

SrtC and its characterized substrates are normally expressed during stationary phase, but phage lysis can only be assayed during exponential phase [18], [24]. Consequently, SrtC substrates, BasH and BasI, were not included in this study.

The eight constructs were transferred to a strain in which *gamR* was deleted. As a control, *gamR* was transferred simultaneously into a Δ*gamR* strain (Table 2). As expected, γ phage lysis was restored upon complementation with GamR [25].

**Table 2:** Repertoire identification.

cwss tail	Wild-type strain		Δ*srtA*		Δ*srtB*		Δ*srtC*		Predicted sortase	Complementa-tion for lysis phenotype with	Identified sortase
test	PL^a^	IB^b^	PL	IB	PL	IB	PL	IB			
GamR	+	+	-	-	+	+	+	+	SrtA	*srtA*: yes^c^	SrtA
Bas B	+	NT	-	NT	+	NT	+	NT	SrtA	*srtA*: yes	SrtA
BasE	+	NT	-	NT	+	NT	+	NT	SrtA	*srtA*: yes	SrtA
BasJ	+	NT	-	NT	+	NT	+	NT	SrtA	*srtA*: yes	SrtA
BasA	+	+	+	-	-	+	+	+	SrtA		SrtA
Bas C	+	NT	+	NT	+	NT	+	NT	SrtA		SrtA^d^
IsdC	+	NT	+	NT	+	NT	+	NT	SrtB		SrtB^d^
BasL	+	+	+	+	+	+	+	+	SrtB		
BasO^e^	-	-	NT	NT	NT	NT	NT	NT	NA	NA	NA

a PL: Phage Lysis test; + : susceptible, – : resistant to phage lysis.

b IB : copurification with the peptidoglycan as assayed by ImmunoBlot; + : copurifies. -: does not copurify.

c : as shown in Davison et al., 2005.

d identified by other authors (BasC, [20]); (IsdC, [23]).

e : Not a cwss protein.

NT, not tested, NA, does not apply.

**Figure 2:**
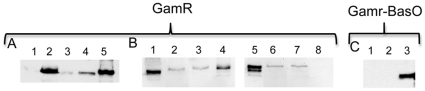
GamR-BasO is not covalently anchored to the peptidoglycan. Different fractions of GamR (A and B) and GamR-BasO (C) producing strains were analyzed by Western blots. **A**. Culture supernatant samples of the parental 7702 strain and is sortase mutant derivatives were loaded; lanes, 1, parental; 2, *srtA*; 3, *srtB*; 4, *srtC*; 5, purified GamR. **B**. Peptidoglycan was purified from the parental 7702 (lanes 1 to 4) and the triple mutant *srtA srtB srtC* (lanes 5 to 8) strains and all the peptidoglycan purification steps were analyzed; lanes, 1 and 5, culture supernatants; 2 and 6, 3 and 7, 4 and 8, peptidoglycan fraction of the purification steps 1, 2 and 3, respectively. These lanes were not all contiguous on the initial gels. **C**. Cells producing GamR-BasO were grown in R-bic and fractionated; lanes, 1, insoluble (cell surface associated) fraction; 2 soluble (cytoplasm) fraction; 3, culture supernatant.

Seven of the 8 strains, in addition to that harboring GamR, displayed susceptibility to the γ phage (Table 2), indicating that these hybrid proteins were accessible to the γ phage. Interestingly, the GamR-BasO hybrid protein did not yield γ phage lysis. We checked that the GamR-BasO fusion protein was indeed produced and determined in which fraction it was present, by western-blotting with anti-GamR antibodies (Fig. 2). When GamR was produced under the control of its native regulatory region, it was, or not, detected, depending on the growth conditions, in the supernatant of the parental strain (Fig. 2A, lane 1, Fig 2B lane 1) and it was present in the cell wall preparations, co-purifying with the peptidoglycan in the parental strain but not the triple mutant strain (Fig. 2B, lane 4 and 8). In contrast to GamR, GamR-BasO was found exclusively in the culture supernatant of the parental strain confirming the absence of anchoring of this chimeric protein (Fig. 2C). This construct served as a negative control; it seemingly validated our assay and indicated that the other seven chimeric proteins were surface exposed.

### Determination of the repertoires of the *B. anthracis* sortases

To determine which sortase anchored each of the remaining eight (seven chimeric and positive control GamR) proteins, we transferred the fusion constructs into the three *gamR* mutant strains, each of which has a deletion of one of the three sortase genes. The susceptibility of the different strains is summarized in Table 2.

As expected, in cells producing under its native regulatory signals GamR with its own LPXTG tail, lysis was observed in both *srtB* and *srtC* mutant strains but not in the *srtA* mutant, in agreement with GamR being anchored only by SrtA (Table 2) [25]. This suggested that the phage system may be used to suggest sortase repertoires. We found that the strains containing the three fusion proteins (GamR-BasB, GamR-BasE and GamR-BasJ) were resistant to lysis in the *srtA* mutant and susceptible in the *srtB* and *srtC* mutant strains, indicating they are anchored by Sortase A, the *in silico* predicted sortase (Table 2). Complementation experiments were carried out to confirm the observed patterns of sortase anchoring for BasB, BasE and BasJ proteins by introducing the wild-type *srt*A gene into *gamR srtA* mutant strains expressing the GamR-BasB, GamR-BasE and GamR-BasJ fusion constructs (see Materials and Methods). Susceptibility to γ phage was restored in all complemented strains confirming that SrtA anchors BasB, BasE and BasJ (Table 2).

In contrast, the four other proteins did not seem to be anchored by, or solely by, the predicted sortase. The *srtB* strain expressing the GamR-BasA fusion protein was resistant to the phage suggesting that BasA could be anchored by SrtB. All the strains producing GamR-BasC, GamR-IsdC and GamR-BasL were lyzed suggesting that the proteins were anchored independently of the present sortases.

### Proteins that in an unexpected manner, or always, yielded γ phage lysis

**Figure 3:**
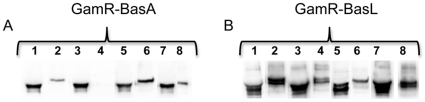
GamR-BasA and GamR-.BasL are covalently anchored to the peptidoglycan. Peptidoglycan was purified from GamR-BasA (A) and GamR-.BasL (B) producing cells grown in R-Bic. Lanes, 1, 3, 5 and 7, culture supernatants; lanes 2, 4, 6 and 8, purified peptidoglycans (step 3); lanes, 1 and 2, parental strains; 3 and 4, *srtA* strains; 5 and 6, *srtB* strains; 7 and 8, *srtC* strains. These lanes were not all contiguous on the initial gels.

The phage lysis assay on the GamR-BasA harboring strains indicated, in contrast with our and others' prediction, a sortase B anchoring ([20], Table 1, Table 2). To assess the anchoring further, the covalent binding of the protein to the peptidoglycan was assayed by immuno-detection (Fig. 3A). GamR, when produced in native conditions, was only weakly found in the culture supernatant of the *srtB* and *srtC* mutant strains (Fig. 2A, lanes 3, 4). When overproduced, GamR chimeric proteins were found in all supernatants, indicating that the overproduction overwhelmed the anchoring capacity (data not shown and Fig. 3A and 3B lanes 1, 3, 5, 7). Consequently, the presence of chimeric proteins in the supernatant could not be used as a criterion for absence of anchoring and the peptidoglycan was purified as in the parental strain and step 3 analyzed (see Fig 2B). The presence of GamR-BasA was sought in the peptidoglycan fraction of the parental and each sortase mutant strain (Fig 3A); to note, GamR present in the peptidoglycan fractions migrated more slowly than that in the supernatant. This could be the consequence of peptidoglycan fragments still anchored to the protein. The absence of signal found in the peptidoglycan fraction from the *srtA* mutant strain and its presence in that of all other strains including the *srtB* mutant (Fig. 3A lane 4 versus lanes 2, 6 and 8) indicated that, in fact, this chimeric protein is anchored by sortase A. The absence of lysis in the *srtA* strain could have been due to a protein production problem in the conditions used. We concluded from these results that BasA is anchored by SrtA (Table 2).

Unexpectedly, the phage susceptibility assay suggested that the GamR-BasC, GamR-IsdC and GamR-BasL fusion proteins were phage-accessible in all mutants. Two possibilities could account for that. The first one is that more than one sortase can catalyze the anchoring of these proteins to the peptidoglycan. The second one is that non-anchored but surface accessible proteins can permit phage lysis. BasC and IsdC have been shown to be sortase A- and sortase B- anchored respectively, and were not further studied [20], [23]. Consequently, our data suggest that chimeric proteins may be present at the surface in sufficient amount and correct conformation to enable phage lysis even when not covalently anchored to the peptidoglycan. Concerning GamR-BasL, and to discriminate between the two hypotheses, immuno-detection experiments were carried out on proteins extracted from peptidoglycan purified from all GamR-BasL producing strains (Fig. 3B). As with overproduced wild-type GamR, the culture supernatant from all strains contained GamR-BasL chimeric protein. In contrast to the situation observed with GamR or GamR-BasA, a signal was found in the peptidoglycan fraction of all strains. However, a weaker signal was found in the *srtB* strain than in the others. These data indicate that this chimeric protein was always found surface-associated and suggest that SrtB may be involved in its anchorage.

## Discussion

We describe here a system in which the anchoring of the *B. anthracis* γ phage receptor, GamR, to the peptidoglycan was used as a screen for determining the ability of sortase proteins to anchor *B. anthracis* cwss proteins to peptidoglycan *in vivo*. Indeed, previous studies suggested that there are 9 cwss proteins in the *B. anthracis* Ames strain whereas our *in silico* analysis suggested that there are 10 cwss proteins (Table 1) [19], [20]. Furthermore, in a first attempt to determine the *B. anthracis* sortase repertoire, Western blots were carried out, as done with *Listeria monocytogenes* or, more recently, with *Streptococcus uberis* for instance [39], [40], with sera raised against some of the cwss proteins. However, the comparison of the protein present in the different fractions did not enable us to define unambiguously the anchoring sortases. Except for GamR, no obvious signal enhancement was detected in the supernatant of a single sortase mutant strain and thorough purification of the peptidoglycan led to too weak signals (Fig 2A and data not shown).

Unfortunately, the chimeric protein-driven phage lysis approach proved to be too sensitive, phage lysis occurring also in strains in which the proteins were not covalently attached to the peptidoglycan. Coupling this system to immuno-detection of overexpressed proteins, we confirmed some already described anchoring and indicated that sortase A, as predicted, anchored BasA and BasJ. Discrimination based on the motif of the consensus sequence seems efficient.

BasL (IsdK, IsdX2), the status of which was not clear, is covalently anchored to the peptidoglycan (Table 1, [20], [26], [27], [41]). However, because GamR-BasL co-purified with the peptidoglycan independently of the sortases synthesized by the producing strains, we cannot definitely ascribe a sortase to BasL anchoring. Either more than one sortase can catalyze it or, less credible, BasL is not a cwss protein and is anchored by another mechanism. The two motifs NPKTG (IsdC) and NSKTA (BasL) share NxK with both *L. monocytogenes* SrtB substrates, namely Lmo2185 (NAKTN) and Lmo2186 (NPKSS) [42]. *L. monocytogenes* SrtB has the capacity to recognize varied amino acids at position 2 of the sorting motif and proline is not absolutely required for substrate recognition. Furthermore, in *B. anthracis*, BasL is in the same operon as IsdC, that is anchored by SrtB, hence simultaneously produced. These proteins belonging to the same physiological pathway, iron metabolism, the involvement of the same sortase is a tempting hypothesis. BasL could yet be, when overproduced, a substrate for SrtA in absence of SrtB: *in vitro*, the LPNTA motif, that is found in BasI normally anchored by SrtC, is a substrate for SrtA [18], [20]. *B. anthracis* thus produces 9 or more probably 10 cwss proteins, 6 of which depend on SrtA, 1 or 2 on SrtB, and 2 on the class D sortase, SrtC, for their anchorage.

Phage lysis was obtained with undiluted phage solution in the *srtA* and in the triple *srtA srtB srtC* mutant strains when GamR was synthesized under the control of its native promoter and rbs [25]. This suggests that even when produced in native quantities GamR proteins are surface exposed in absence of covalent anchoring to the peptidoglycan. This is in contrast to the situation observed in other bacteria, in which sortase A deleted mutant do not display, at their surface, sortase A substrate, such as InlA in *L. monocytogenes,* Protein A in *Staphylococcus aureus* [39], [43]. This apparent difference could also be a consequence of the protein quantities synthesized by the bacteria and the detecting methods used. Low amounts of unprocessed form of surface proteins may be undetectable by immuno-reaction. That GamR is surface exposed in *B. anthracis srtA* mutant strains could be a consequence of *B. anthracis* lacking efficient LPXTGase activity. LPXTGase are non-ribosomally synthesized enzymes that cleave LPXTG motifs with a catalytic activity higher than that of sortase [44], [45]. *B. anthracis* could be devoid of LPXTGase or *B. anthracis* LPXTGase could be less active than that of *Streptococcus pyogenes* [45]. This could be a selected consequence of *B. anthracis* SrtA hydrolyzing the LPXTG sorting signal 40 times slower than *S. aureus* SrtA enzyme [17].

Although *srtA* and *srtB* strains displayed attenuated growth in macrophage-like cell line, no virulence defect has been observed with sortase mutant strain in various animal models [18], [20], [21], [22], [46]. A possible explanation is that due to the high level of redundancy observed in *B. anthracis* genome, important functions are not encoded by a single gene, harbored by a single protein [38]. However, given functions may require a precise localization, including surface-association. In that respect, the permanent presence of cwss membrane-associated, non-covalently anchored, proteins at the surface of *B. anthracis* may satisfy this need.

In conclusion, the method relying on phage sensitivity yielded a conclusion similar to the prediction for four sortase A anchored substrates. However, it proved too sensitive for defining unambiguously the repertoire of the sortases, phage lysis being, for some chimeric proteins, observed in all sortase mutant strains. It would be worth testing whether changing the promoter region of the chimeric genes for a weaker one could alleviate this drawback. In one occurrence, the result obtained was misleading. Immunoblot experiments carried out on purified peptidoglycan preparations represent efficient complementary tests and should be considered for chimeric proteins for which unexpected results are obtained.

## Supporting Information

Table S1 ***B. anthracis strains*** **, plasmids, and oligonuceotides used in this study.**
(DOCX)
