# Supplementary material for: Characterization of the Sortase Repertoire in Bacillus anthracis
Source: PLoS One. 2011 Nov 4;6(11):e27411. doi: 10.1371/journal.pone.0027411 (PMC3208642; doi:10.1371/journal.pone.0027411)
Supplement: Table S1 — B. anthracis strains , plasmids, and oligonuceotides used in this study. (DOCX) [file pone.0027411.s001.docx]

Table S1: *B.* *anthracis strains*, plasmids, and oligonuceotides used in this study

| Strain, plasmid,or oligonucleotide | Genotype or description | Source/  construction | |
| --- | --- | --- | --- |
| ***Bacillus anthracis*** |  |  | |
| 7702 | Sterne strain; pXO1^+^ | Laboratory stock | |
| 7SBON30 | 7702 ∆*srtA* | [[1](#_ENREF_1)] | |
| 7SBTR30 | 7702 ∆*srtB* | [[1](#_ENREF_1)] | |
| 7SBTO30 | 7702 ∆*srtC* | [[1](#_ENREF_1)] | |
| 7G | 7702 ∆*gamR* | This work | |
| 7AG | 7702 ∆*srtA* ∆gamR | This work | |
| 7BG | 7702 ∆*srtB* ∆gamR | This work | |
| 7CG | 7702 ∆*srtC* ∆gamR | This work | |
| 7GBasA | 7G strain g*amR’-‘basA*+ | This work | |
| 7AGBasA | 7AG strain g*amR’-‘basA*+ | This work | |
| 7BGBasA | 7BG strain g*amR’-‘basA*+ | This work | |
| 7CGBasA | 7CG strain g*amR’-‘basA*+ | This work | |
| 7GBasB | 7G strain *gamR’-‘basB*+ | This work | |
| 7AGBasB | 7AG strain *gamR’-‘basB*+ | This work | |
| 7BGBasB | 7BG strain *gamR’-‘basB*+ | This work | |
| 7CGBasB | 7CG strain *gamR’-‘basB*+ | This work | |
| 7AGCABasB | 7AG strain *gamR’-‘basB*+, *srtA*+ | This work | |
| 7GBasC | 7G strain *gamR’-‘basC*+ | This work | |
| 7AGBasC | 7AG strain *gamR’-‘basC*+ | This work | |
| 7BGBasC | 7BG strain *gamR’-‘basC*+ | This work | |
| 7CGBasC | 7CG strain *gamR’-‘basC*+ | This work | |
| 7GGamR | 7G strain *gamR*+ | This work | |
| 7AGGamR | 7AG strain *gamR*+ | This work | |
| 7BGGamR | 7BG strain *gamR*+ | This work | |
| 7CGGamR | 7CG strain *gamR*+ | This work | |
| 7GBasE | 7G strain *gamR’-‘basE*+ | This work | |
| 7AGBasE | 7AG strain *gamR’-‘basE*+ | This work | |
| 7BGBasE | 7BG strain *gamR’-‘basE*+ | This work | |
| 7CGBasE | 7CG strain *gamR’-‘basE*+ | This work | |
| 7AGCABasE | 7AG strain *gamR’-‘basE*+, *srtA*+ | This work | |
| 7GBasJ | 7G strain *gamR’-‘basJ*+ | This work | |
| 7AGBasJ | 7AG strain *gamR’-‘basJ*+ | This work | |
| 7BGBasJ | 7BG strain gamR’-‘*basJ*+ | This work | |
| 7CGBasJ | 7CG strain *gamR’-‘basJ*+ | This work | |
| 7AGCABasJ | 7AG strain *gamR’-‘basJ*+, *srtA*+ | This work | |
| 7GIsdC | 7G strain *gamR’-‘IsdC*+ | This work | |
| 7AGIsdC | 7AG strain *gamR’-‘IsdC*+ | This work | |
| 7BGIsdC | 7BG strain *gamR’-‘IsdC*+ | This work | |
| 7CGIsdC | 7CG strain *gamR’-‘IsdC*+ | This work | |
| 7GBasL | 7G strain *gamR’-‘basL*+ | This work | |
| 7AGBasL | 7AG strain *gamR’-‘basL*+ | This work | |
| 7BGBasL | 7BG strain *gamR’-‘basL*+ | This work | |
| 7CGBasL | 7CG strain *gamR’-‘basL*+ | This work | |
| 7GBasO | 7G strain *gamR’-‘basO*+ | This work | |
|  |  |  | |
| **Plasmid** |  |  | |
| pCR2.1 | cloning vector | Invitrogen | |
| pGEM-T-easy | cloning vector | Qiagen | |
| pQE30 | Expression vector | Qiagen | |
| pAT21 | kanamicin resistance cassette-harboring plasmid | [[2](#_ENREF_2)] | |
| pAT∆S28 | non replicative plasmid, Spc^R^ | [[3](#_ENREF_3)] | |
| pPPA40 | replicative vetor harboring the *pag* promoter region | [[4](#_ENREF_4)] | |
| pGAR10 | pGem-T-easy carrying the *gamR* gene | [[1](#_ENREF_1)] | |
| pGARK20 | pGem-T-easy carrying the inactived *gamR* gene, KanR | This work | |
| pGARK30 | pAT∆S28 carrying the inactived *gamR* gene, KanR | This work | |
| pGamR10 | pCR2.1 carrying the *gamR* gene nucleotides 1 to 1787 | This work | |
| pGamR30 | pPPA40 carrying the gamR gene nucleotides 1 to 1787 | This work | |
| pBasA10 | pCR2.1 carrying nucleotides 2189 to 2324 of the *basA* gene | This work | |
| pBasA20 | pGamR10 digested with AgeI/XbaI followed in frame by pBasA10 insert | This work | |
| pBASA30 | pPPA40 carrying the *gamR’-‘basA* fusion | This work | |
| pBasB10 | pCR2.1 carrying nucleotides 2778 to 3047 of the *basB* gene | This work | |
| pBASB20 | pGamR10 digested with AgeI/SacI followed in frame by pBasB10 insert | This work | |
| pBASB30 | pPPA40 carrying the *gamR’-‘basB* fusion | This work | |
| pBasB40 | pPPA40 carryng the *gamR’-‘basB* fusion in operon with the *srtA* gene | This work | |
| pBasC10 | pCR2.1 carrying nucleotides 1789 to 2038 of the *basC* gene | This work | |
| pBasC20 | pGamR10 digested with AgeI/XbaI followed in frame by pBasC10 insert | This work | |
| pBasC30 | pPPA40 carrying the *gamR’-‘basC* fusion | This work | |
| pBasE10 | pCR2.1 carrying nucleotides 974 to 1192 of the *basE* gene | This work | |
| PBasE20 | pGamR10 digested with AgeI/SacI followed in frame by pBasE10 insert | This work | |
| pBasE30 | pPPA40 carrying the *gamR’-‘basE* fusion | This work | |
| pBasE40 | pPPA40 carryng the *gamR’-‘basE* fusion in operon with the *srtA* gene | This work | |
| pBasJ10 | pCR2.1 carrying nucleotides 3183 to 3694 of the *basJ* gene | This work | |
| pBasJ20 | pGamR10 digested with AgeI/SacI followed in frame by pBasJ10 insert | This work | |
| pBasJ30 | pPPA40 carrying the *gamR’-‘basJ* fusion | This work | |
| pBasJ40 | pPPA40 carryng the *gamR’-‘basJ* fusion in operon with the *srtA* gene | This work | |
| pIsdC10 | pCR2.1 carrying nucleotides 545 to 721 of the *isdC* gene | This work | |
| pIsdC20 | pGamR10 digested with AgeI/SacI followed in frame by pIsdC10 insert | This work | |
| pIsdC30 | pPPA40 carrying the *gamR’-‘isdC* fusion | This work | |
| pBasL10 | pCR2.1 carrying nucleotides 2495 to 2731 of the *basL* gene | This work | |
| pBasL20 | pGamR10 digested with AgeI/XbaI followed in frame by pBasL10 insert | This work | |
| pBasL30 | pPPA40 carrying the *gamR’-‘basL* fusion | This work | |
| pBasO10 | pCR2.1 carrying nucleotides 601 to 877 of the *basO* gene | This work | |
| pBasO20 | pGamR10 digested by AgeI/SacI followed in frame by pBasO10 insert | This work | |
| pBasO30 | pPPA40 carrying the *gamR’-‘basO* fusion | This work | |
| p3367 | pGEM-T-easy carrying the *gamR* ORF | This work | |
| pQE3367 | pQE30 carrying the *gamR* ORF in frame with the His-tag | This work | |
|  |  | |  |
| **Oligonucleotide** |  | |  |
| Prebas-5' | GTCGCCAACCTTTTCGGTCTTTTGGAAAGA | |  |
| Bas5’ | GGATCCGTCATGGGGACAAGTAGAAGCTGAAAC | |  |
| Bas-3' | AAGCTTAGCTGCTTTACGTCTCCATAATACATATGC | |  |
| BasA-5' | ACCGGTAACTGTAAGTGAGAACAAAGAAGAACGTGATTTACC | |  |
| BasA-3' | AAGCTTTTATCTATTCGCTTTCTTTCTTCTAAATAA | |  |
| BasB-5' | ACCGGTAGAAAAAGGACAAGGTACCTCTCATGCTCAACAGCTTCCAGCC | |  |
| BasB-3' | GAATTCGTACCAAAAGGCACAGCAACACGCGCTGAATCCG | |  |
| BasC-5' | ACCGGTGCCAATGCAACAACCTAGTACTCATAATGAAGTGCGTTTACC | |  |
| BasC-3' | CAGAATTCTTTTATCACGACGTTGCACTTGCAAAGATTACCC | |  |
| GamR-5' | CATATGGCAATGTTAAAAAAATGGCTGCTTACGTCGTTAATGGC | |  |
| GamR-3' | AAGCTTAGCTGCTTTACGTCTCCATAATACATATGC | |  |
| BasE-5' | ACCGGTTACTACAAATGCAAACAATAATGCTAGAGAACTTCC | |  |
| BasE-3' | GTTAATATAAAGGAAAGGGCCAACCGTACATGCGGTTGGCC | |  |
| BasJ-5' | ACCGGTAGCTGTTACTAAGCAAGAAGAAAGTAAGAAATCACTAGG | |  |
| BasJ-3' | CAATATGAAAAGGCACCTTTTCTATAAGTTCCTTCC | |  |
| IsdC-5' | ACCGGTGAGTAAGGAAACAGCTAAAGAAGTGAAC | |  |
| IsdC-3' | GGCTTTCAAAATACCTATTCATTCTAGACG | |  |
| BasL-5' | ACCGGTAGCTACAAATAATGATGCGAAAAAAGAAAAAAACTCAAAAACAGC | | |
| BasL-3' | GCCATTAGTACACTTGCAATTTTCTTCACTCTCAATCACCACTCC | |  |
| BasO-5' | ACCGGTTGCAAATGTAAAAGAACTTTTATTAACGCACTTACCGC | |  |
| BasO-3' | CCAATAATAATCGAAGGCTCATTAATGTAGAACAGTAAATATGTTTCG | |  |
| CA1-5' | GAGCTCGGAGGAAAAATATAAATGAATAAGCAAAGAATTTATAGTATAGTAGCAATCCTCC | | |
| CA2-3' | GAATTCTTATTTCTTCGCCTTCGTTCCTACTAAATCACCAGC | |  |

1. Davison S, Couture-Tosi E, Candela T, Mock M, Fouet A (2005) Identification of the *Bacillus anthracis* (gamma) phage receptor. J Bacteriol 187: 6742-6749.

2. Trieu-Cuot P, Courvalin P (1983) Nucleotide sequence of the Streptococcus faecalis plasmid gene encoding the 3'5"-aminoglycoside phosphotransferase type III. Gene 23: 331-341.

3. Namy O, Mock M, Fouet A (1999) Co-existence of *clpB* and *clpC* in the *Bacillaceae*. FEMS Microbiol Lett 173: 297-302.

4. Candela T, Mock M, Fouet A (2005) CapE, a 47-amino-acid peptide, is necessary for *Bacillus anthracis* polyglutamate capsule synthesis. J Bacteriol 187: 7765-7772.
